# Implication of Topical Steroids in the Onset of Osteoporosis

**DOI:** 10.1155/2018/4802172

**Published:** 2018-08-29

**Authors:** Asmaa Adel Milyani, Abdulmoein Eid Al-Agha

**Affiliations:** ^1^Faculty of Medicine, King AbdulAziz University, P.O. Box 80215, Jeddah, Saudi Arabia; ^2^Paediatric Department, King AbdulAziz University Hospital, P.O. Box 80215, Jeddah 21589, Saudi Arabia

## Abstract

The systemic bioavailability of steroids has long been implicated as a cause for osteoporosis (OP); however, much less is known about the effect of topical steroids on bone homeostasis. This is a case of an 11-year-old male who is a known case of generalised pustular psoriasis for 8-year duration with frequent exacerbations controlled with topical betamethasone dipropionate. He presented with generalised progressive bone pain and positive history of bone fracture. The diagnosis of OP was established on the results of DEXA, which were −2.7 SD and −2.4 SD for the lumbar spine and whole body, respectively. Although the cutoff value is the same (<−2 SD) in children, the definition of OP is more reliant on the densitometry *Z* score, as opposed to adults, who are approached using the *T* score. The element of psoriasis poses a risk for the development of OP due to the presence of a chronic inflammatory disease state that increases bone turnover. Furthermore, the compromised skin barrier and associated vasodilation seen in psoriasis enhance the absorption of topically applied agents and increase their bioavailability. Children are a targeted risk group as they are more vulnerable to the manifestation of systemic adverse affects of topically applied steroids as a result of their increased ratio of total surface area relative to their body weight and slower drug metabolism. We recommend that children undergoing long-term topical steroid therapy be screened for OP with the consideration of instituting prophylactic treatment especially in those suffering from chronic inflammatory disease states.

## 1. Introduction

Osteoporosis in children can either be due to primary causes, of which the most common is osteogenesis imperfecta, or secondary causes, of which the most common is attributed to prolonged glucocorticoid usage. Clinically, osteoporosis in children presents with bone fracture either in a child known to suffer from a chronic disease or in a previously healthy child suffering from mild osteoporosis [1]. While the deleterious effect of low-dose systemic corticosteroids on bone homeostasis has long been established [2], a considerable degree of uncertainty is still yet to be unraveled as to the effect of topical corticosteroids. Children in particular are more prone to the development of systemic adverse effects relating to topically applied corticosteroids due to their higher ratio of total body surface area to total body weight [3]. This brings attention to the variables that would determine the likelihood of osteoporosis in children treated with topical steroids. This also includes the total area medicated, frequency of application, amount and potency of the associated steroid ointment, duration of therapy, and the integrity of the skin barrier that is exposed to the topical steroid [4]. In psoriatic skin, the impairment of the skin barrier facilitates cutaneous penetration independent of potency, and alongside psoriatic vessels, the concomitant vasodilation increases systemic bioavailability. With long-term use, especially in severe cases where a more generalised presentation is involved and a larger surface area is covered with concentrated steroids in order to achieve dermatological therapeutic targets, osteoporosis, alongside other systemic side effects of steroids, is therefore anticipated [5]. While osteoporosis in the paediatric age group carries little mortality, it does bring about a considerable burden of morbidity, especially due to pain, growth impairment, and long-term sequelae [1]. We report a case of an 11-year-old male with osteoporosis in the setting of prolonged topical corticosteroid use for treatment of his generalised pustular psoriasis.

## 2. Case History

**Figure 1:**
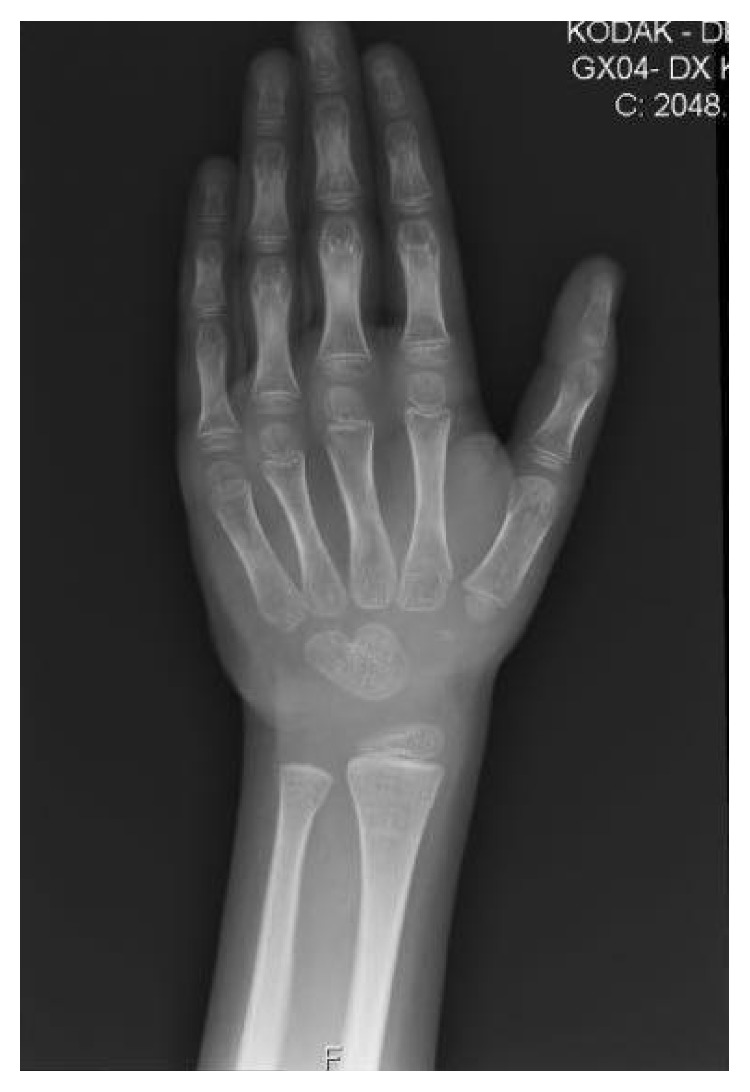
Significant osteopenia noted on left hand radiograph and a reduction in bone age of 1 year based on the metacarpal and phalangeal epiphysis.

**Figure 2:**
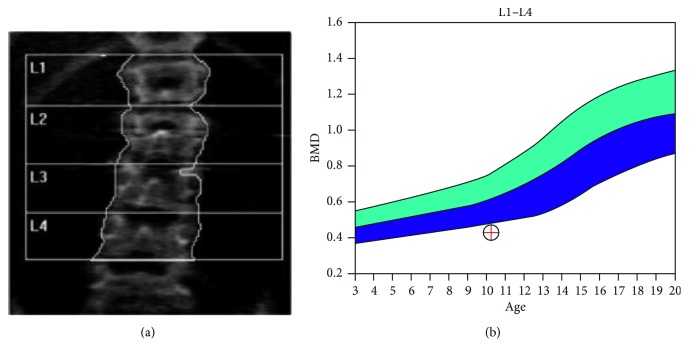
Bone mineral density scan of the vertebra (L1–L4) showing an age-matched *Z* score of −2.7.

**Figure 3:**
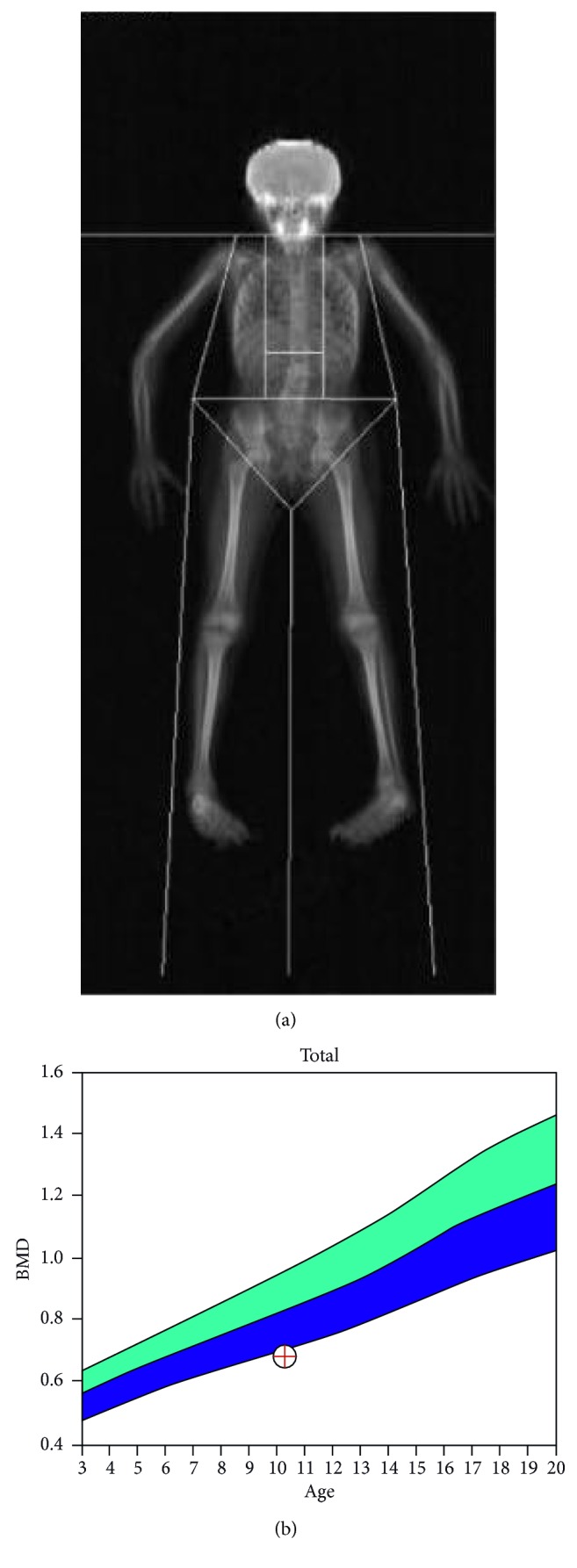
A bone mineral density scan of the whole body showing an age-matched *Z* score of −2.4.

An 11-year-old Saudi boy had presented with severe generalised bone pain and positive history of a left wrist fracture 6 months ago following mild trauma. He is a known case of generalised pustular psoriasis for 8-year duration maintained on daily topical calcipotriol, a vitamin D derivative, and intermittent use of topical betamethasone dipropionate during flare-ups. The bone pain had initially begun three years ago concentrating most in his lower limbs and elicited only with exertion. However, it was becoming progressively more severe in character and generalised in nature, further exacerbated with movement, and partially relieved with rest and immobility, therefore affecting his quality of life by limiting his ability as a child to run, jump, climb stairs, and get up from a seated position. He had been diagnosed with psoriasis when he was 3 years of age upon the results of a skin biopsy. Since then, he would experience a single exacerbation episode of severe generalised pustular psoriasis on an annual basis during wintertime, for which he would require hospitalisation for hydration and administration of antibiotics. During each exacerbation, he was prescribed a topical corticosteroid of betamethasone dipropionate to apply over all affected areas, which would include his face, arms, trunk, and lower limbs. On average, the duration of his topical steroid therapy was restricted to two to three weeks per year for the past 8 years. For the past year prior to his presentation, his condition had progressed with an increase in the rate of his relapses, suffering three exacerbations in the same year. Therefore, he had necessitated more frequent and generous applications of topical betamethasone lasting two to three weeks per flare-up. Upon physical examination, his height was recorded at 124 cm (<3rd percentile) and weight at 28 kg (6th percentile), with a BMI of 18.2 (66th percentile). Total body surface area (BSA) was calculated at 0.98 m^2^. An X-ray of his left wrist showed delayed bone age (Figure 1). A laboratory bone profile was ordered and was compatible with the picture of osteoporosis (Table 1). The diagnosis of osteoporosis was made when the DEXA scan revealed a *Z* score of −2.7 and −2.4 for the lumbar spine (Figure 2) and whole body (Figure 3), respectively. The patient was consequently started on a trimonthly cyclic therapy of intravenous bisphosphonates in the form of zoledronic acid to treat his osteoporosis. Informed consent for publication had been obtained from the legal guardians of the patient prior to writing this paper.

## 3. Discussion

The definition of osteoporosis varies in the paediatric population from that applied to the adult population in the sense that it is more reliant on the densitometry *Z* score than the *T* score usually resorted to in adults. The criteria for diagnosis necessitate the prior or current presence of a pathological fracture in the setting of a low bone mineral content or density. It suffices to say that a *Z* score of a −2 standard deviation would define the presence of osteoporosis [6, 7]. It is more common for children to experience secondary causes of osteoporosis rather than primary, with significant emphasis on the involvement of glucocorticoid usage, marking it to be the most common cause of osteoporosis in children. Topical steroids are one of the most commonly used medications in dermatology. They were designed to deliver therapeutically effective doses to target organs inducing the least possible systemic side effect. However, data regarding the onset of systemic features resulting from topical steroid use began to accumulate gradually, indicating percutaneous absorption. Further studies were directed towards the investigation of contributing factors that predetermine the extent of absorption. Age was found to be an important risk factor due to the slower rate of drug metabolism seen in children, with frequency of application and duration of therapy reinforcing the likelihood of developing side effects [4]. In this case, the patient had been exposed to topical steroid use on an annual basis ever since he was 3 years of age, with an increase in frequency and amount of usage during his past year due to more frequent relapses. This highlights the significant risk of systemic bioavailability that accompanies the use of so much as even small amounts of topical steroids in children especially when over a long duration. One study had showed that over 40 cases of iatrogenic Cushing syndrome was documented with topical steroid use, with the majority of cases manifesting in children and few in adults, with psoriasis being the second most common indication in the former group [8]. In the abovementioned study, the most common used steroids were pertaining to potent categories, clobetasol propionate and betamethasone, which is in concordance with our study, as the ointment used was betamethasone. Knowledge of topical corticosteroid potency is vital to ensure appropriate use. Although corticosteroids have favourable tolerability profiles when used within routine therapeutic ranges, excessive exposure to very potent corticosteroids, such as betamethasone, is associated with an increased risk of cutaneous and systemic side effects [9]. While osteoporosis is a well-recognised side effect of systemic and inhaled corticosteroid, varying degrees of bone mineral density loss has been witnessed in cases of topical steroid use, especially in association with psoriasis [10, 11]. This may be explained by the fact that another important cause of secondary osteoporosis is an underlying chronic systemic inflammatory disease, which by role contributes to defective bone metabolism as a result of increased circulating inflammatory cytokine levels [1]. However, this patient had presented while in remission, and therefore, inflammatory markers such as erythrocyte sedimentation rate, C-reactive protein, interleukins, and tumour necrosis factor were negative for the past six months. One study had ventured to investigate the bone mineral density of patients with psoriasis, attempting to find a correlation between severity index and duration of disease [12]. The results had come back confirming a significant relationship (*p*=0.04) between disease duration and loss of bone mineral density. This patient had been suffering from psoriasis for duration of 8 years before his complaints of bone pain began alongside his wrist fracture. The level of physical activity was described to be the same as of any other child, according to his mother, but he had been progressively becoming more sedentary as his playtime was being limited by exacerbated bone pain. Other causes of bone pain, such as joint laxity, and primary bone disorders were excluded. One case history was found reporting a similar incidence of osteoporosis in relation to topical steroid use, with evidence of systemic bioavailability evident in suppression of the hypothalamic-pituitary axis, affecting both growth and puberty [3]. In resemblance, the patient was an adolescent male who suffered from generalised pustular psoriasis and was prescribed a potent topical steroid, clobetasol propionate. However, the duration of psoriasis, 5 years, was less than our case and had recruited a much more frequent and unsupervised application of the ointment. Furthermore, his growth parameters had exhibited short stature with a height below the 3rd percentile for age and gender alongside obesity with his weight above the 97th percentile. In our case, the patient's weight is slightly above average for his age and gender, 66th percentile, but is similarly suffering from short stature as his height is as well beyond the 3rd percentile. Growth is impaired by glucocorticoids due to the suppression of the hypothalamic-pituitary axis leading to a decrease in secretion of growth hormone-releasing hormone (GHRH) and growth hormone [4]. This most logically accounts for the short stature found in both patients. However, upon performing a Synacthen stimulation test, the adrenal axis was found to be still within normal functioning. Thereby, and although the patient manifested growth parameters indicative of growth retardation, the hypothalamic-pituitary-axis was concluded to be intact. Calcipotriol is a nonsteroidal alternative in the treatment of mild to moderate plaque-type psoriasis and has utility as monotherapy, as well as in combination with topical steroids, and can be used to reduce the frequency or steroidal therapy and associated adverse effects. However, corticosteroids still remain the mainstay topical treatment of psoriasis, as they have anti-inflammatory and antiproliferative properties and reduce erythema, scaling, and pruritus. Therefore, it is recommended to use lower potency preparations in areas with higher absorption such as the face, mucosal membranes, genitals, and intertriginous areas, preserving the higher potency preparations for thick hyperkeratotic areas such as the palms and soles [4]. Unfortunately, in this case, the patient had been using a single high potent preparation for all of his body, which could have contributed to the increased systemic bioavailability of the drug.

In conclusion, systemic side effects of topical glucocorticoid use, osteoporosis in particular, should be anticipated in the paediatric population due to their predisposition as a result of their decreased ratio of total surface area to total body weight. Therefore, we recommend careful consideration for a judicious use of topical steroids in children, and if found necessary, the screening for osteoporosis and consequent institution of prophylactic management.

## Figures and Tables

**Table 1:** Bone profile.

	Result	Reference range
Calcium (mmol/L)	2.17	2.2–2.7
Phosphate (mmol/L)	1.60	1.07–1.74
Alkaline phosphatase (U/L)	198	100–390
Parathyroid hormone (Pmol/L)	2.9	1.18–8.43
25-OH vitamin D3 (nmol/L)	101	75–250

## References

[1] Uziel Y., Zifman E., Hashkes P. J. (2009). Osteoporosis in children: pediatric and pediatric rheumatology perspective: a review. *Pediatric Rheumatology*.

[2] Van Staa T. P., Leufkens H. G. M., Cooper C. (2002). The epidemiology of corticosteroid-induced osteoporosis: a meta-analysis. *Osteoporosis International*.

[3] Sahana P. K., Sarma N., Sengupta N., Somani P. S. (2015). A florid case of iatrogenic Cushing’s syndrome induced by topical steroid with osteoporosis and hypogonadism. *Indian Journal of Dermatology*.

[4] Dhar S., Seth J., Parikh D. (2014). Systemic side-effects of topical corticosteroids. *Indian Journal of Dermatology*.

[5] Uva L., Miguel D., Pinheiro C. (2012). Mechanisms of action of topical corticosteroids in psoriasis. *International Journal of Endocrinology*.

[6] Lewiecki E. M., Gordon C. M., Baim S. (2008). Special report on the 2007 adult and pediatric Position Development Conferences of the International Society for Clinical Densitometry. *Osteoporosis International*.

[7] Bishop N., Braillon P., Burnham J. (2008). Dual-energy X-ray aborptiometry assessment in children and adolescents with diseases that may affect the skeleton: the 2007 ISCD pediatric official positions. *Journal of Clinical Densitometry*.

[8] Hengge U. R., Ruzicka T., Schwartz R. A., Cork M. J. (2006). Adverse effects of topical glucocorticosteroids. *Journal of the American Academy of Dermatology*.

[9] Queille-Roussel C., Bang B., Clonier F., Lacour J. P. (2016). Enhanced vasoconstrictor potency of the fixed combination calcipotriol plus betamethasone dipropionate in an innovative aerosol foam formulation vs. other corticosteroid psoriasis treatments. *Journal of the European Academy of Dermatology and Venereology*.

[10] Nymann P., Kollerup G., Jemec G. B. E., Grossmann E. (1996). Decreased bone mineral density in patients with pustulosis palmaris et plantaris. *Dermatology*.

[11] Nielsen N. V. (1978). Glaucoma induced by application of corticosteroids to the periorbital region. *Archives of Dermatology*.

[12] D’Epiro S., Marocco C., Salvi M. (2014). Psoriasis and bone mineral density: implications for long-term patients. *Journal of Dermatology*.

